# The art of medicine: The end, and what comes after

**DOI:** 10.1016/S0140-6736(25)00044-3

**Published:** 2025-01-18

**Authors:** Laura Salisbury, Dora Vargha, Debora Diniz, Luciana Brito, Osman Sankoh, Haja Ramatulai Wurie, Regina Mamidy Yillah, Sharifah Sekalala, Caroline Dubois, Emily Ying Yang Chan, Yureshya Perera, Ruth Ogden, Patricia Kingori

**Affiliations:** Department of English and Creative Writing, University of Exeter, Exeter EX4 4QH, UK; Lived Experiences and Aftermaths of Diseases, Disasters and Drugs in Global Health Project, Ethox Centre, Oxford Population Health, University of Oxford, Oxford, UK; Lived Experiences and Aftermaths of Diseases, Disasters and Drugs in Global Health Project, Ethox Centre, Oxford Population Health, University of Oxford, Oxford, UK

In March, 2024, Paul Alexander, the last person in the world living with an iron lung, died. Alexander had contracted poliomyelitis in 1952 when he was 6 years old, and from that point onwards was only able to move his head, neck, and mouth. Iron lungs became a symbol of polio during the epidemic outbreaks of the 1950s, when they were widely used to save the lives of patients with respiratory paralysis during the acute phase of the disease. Iron lungs are now staple exhibition pieces in history of medicine museums, as global polio vaccination efforts have put a stop to major epidemic waves. However, Alexander reminds us that polio was far from being over in the mid-20th century. His life, entangled with a technology viewed by most as an anachronism, and the experiences of hundreds of thousands of people living on with the disease, shape stories that run counter to straightforward heroic accounts of medical progress and disease elimination and eradication. These lived experiences challenge narratives of epidemics that are structured according to the possibility of marking a clearly discernible end. As we can see in the re-emergence of polio in Gaza in 2024, disease eradication efforts themselves need constant attention and careful re-evaluation. Endings are fragile, requiring ongoing maintenance; they can easily break down in the face of ongoing privations, conflict, and new lived experiences of atrocity.

For whom is an epidemic over, then, when mass transmission stops; and who is left out when an ending is declared? Many of the 20th century’s most trumpeted achievements in global health have been structured around bringing health crises, and even diseases themselves, to an end. Nevertheless, in the 21st century infectious diseases endure. The world lives on with HIV/AIDS; re-emergences of cholera, plague, polio, Ebola virus disease, and Marburg virus disease; a high burden of multidrug-resistant tuberculosis; new strains of avian and swine influenza viruses; outbreaks of SARS, MERS, Zika virus, and mpox; and the emergence of SARS-CoV-2 that led to the COVID-19 pandemic.

The models that underpin many ideas of global health understandably focus on identifying problems to be treated or mitigated; and implicit within them is the hope of bringing situations or conditions to an end. In the case of epidemics, narratives of understanding and intervention can rely heavily on mobilising a dramaturgic form that moves in a linear way from crisis to response, interventions, and then to closure. This approach shapes ideas of appropriate action and the expectations of what the future might bring. But when we attend to more biographical, historical, or sociological notions of living epidemics, and take account of broader political and social contexts, we can see that a biomedical timeline of a beginning, peak, and ending sits in tension with the repetitions, reversals, suspensions, and endurances of time as it is lived.

**Figure:**
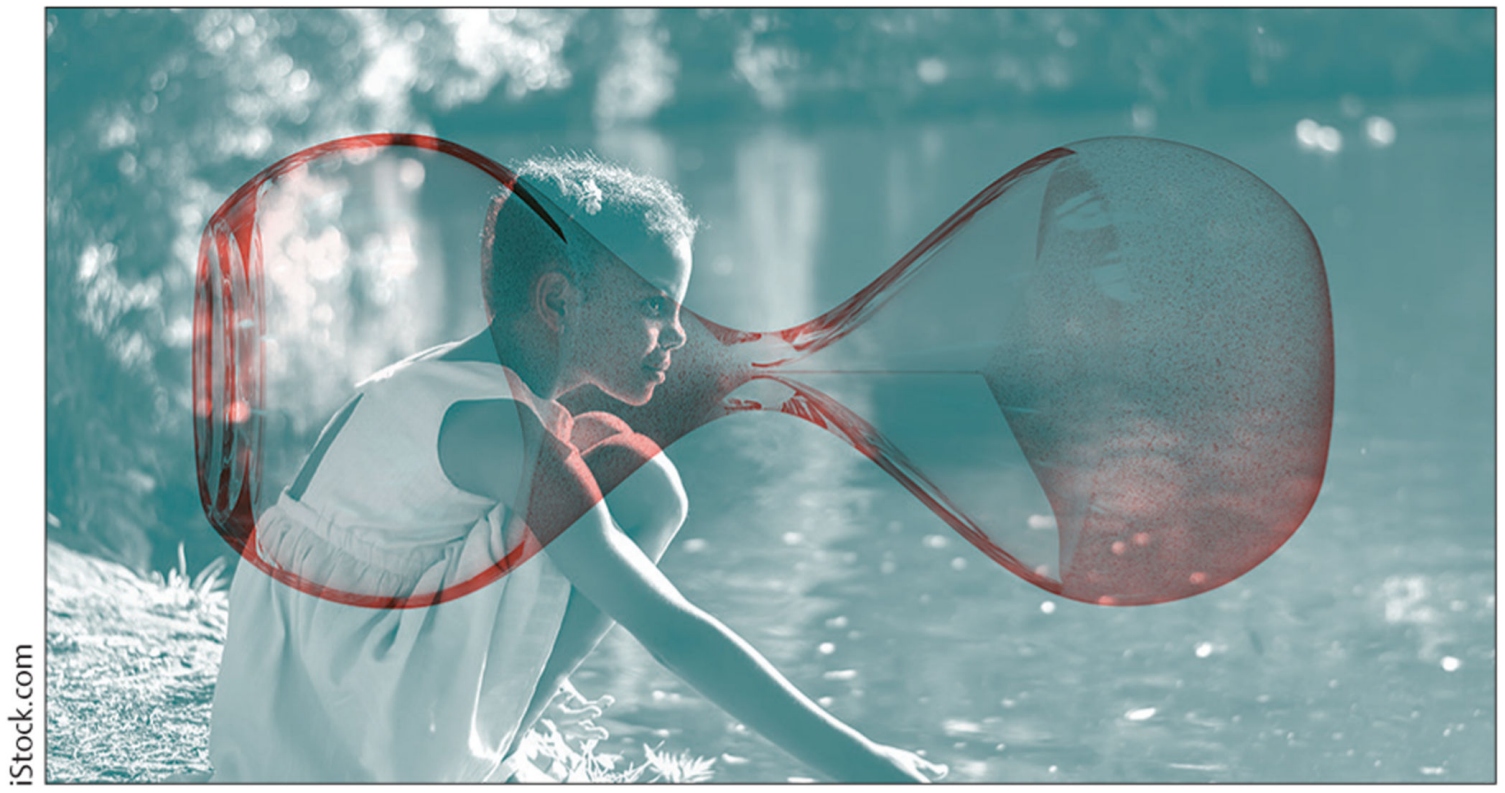


The process of identifying and declaring an end nevertheless has personal, social, legal, and political significance. In May, 2023 the Director-General of WHO, Tedros Adhanom Ghebreyesus, declared, “with great hope”, that COVID-19 was over as a Public Health Emergency of International Concern (PHEIC); but he noted that this end was not the end. He explicitly paid attention to the fact that thousands of people around the world were still fighting for their lives in intensive care, in many parts of the Global South vaccination initiatives were still ongoing, and millions of people will be living on with the effects of post-COVID-19 conditions. The WHO Director-General emphasised that “a lack of coordination, a lack of equity and a lack of solidarity” had led to failures that should never be repeated, especially in the equitable distribution of vaccines and diagnostics. So, although declaring an end seemed to suggest a return to the status quo, it also signalled a movement into a time of reckoning, which included a restatement and reinvigoration of WHO’s mission. To speak of an end to this emergency also required a call for justice and health equity.

Declaring an ending to a health emergency is always a polysemic message, then. Whether the endings used in global or local policy making are official declarations, legal settlements, or epidemiological concepts, they play a part in mobilising resources, providing hope, and shaping aims towards which people might work. But endings can also interrupt resource flow and modify behaviour negatively. The announcement in 2016 by then WHO Director-General Margaret Chan that the Ebola virus disease outbreak was no longer a PHEIC was followed by a reduction of international funding to affected west African nations, as the ongoing needs of communities impacted by Ebola and overstretched health systems were deprioritised. When particular figurations of the end dominate, either as something to be achieved, or even staved off, there is a narrowing of attention to what is imagined as lying ahead that can reproduce current power structures and inflict or perpetuate forms of injustice.

Crises, emergencies, and disasters are all concepts that work with time, shaping and ordering multiple events and experiences into patterns that render them meaningful. If we want to aim for global equity in health, it is crucial to recognise, understand, and critically approach how dominant temporal concepts produce priorities that underwrite health interventions, or the lack thereof. For example, the actions needed to bring a health crisis to an end are sometimes framed in militaristic terms; but this takes little account of how a decisive battle, or the end of a military campaign, does not mean an end to the lived experience of war. Medieval Eurocentric narratives of the “end times” that focus on a prophetic structure of apocalypse and redemption can recur in popular cultural representations of disaster, influencing expectations of how interventions play out and ends can be achieved. But “crisis”, which is derived from the Greek *krinein* (to decide, separate) and the Hippocratic idea of a moment of medical diagnosis, emphasises how human judgement can intervene to shape the future. Some historians have argued that Eurocentric modernity turned from medieval temporalities of prophecy to an Enlightenment belief in crisis and prognosis, in which political and economic activity could organise time and intervene in events. The idea of human action as the motor of historical time was further consolidated during the 19th century through the notion of progress, which implied that ongoing time could be given a direction and orientation. Concepts of crisis and progress may work to synchronise perspectives on time, and the multiple durations, rhythms, and tempos of lived experience; but because experience is diverse and ideas of time from different cultures and traditions run concurrently, measures required to achieve an end often play out paradoxically.

Despite the dominance of temporalities in which a present intervention is framed as overcoming the past to make way for a better and more promising future, lived experiences of emergencies can be more cyclical than linear. Endings intertwine, as hazard scenarios overlap with and magnify one another. Environmental disasters or new resistances to previously effective drugs frequently accompany disease outbreaks; the “slow violence” of environmental degradation intersects with the uncertain trajectories of diseases, interrupting end-oriented imaginaries of cure. Communities can be caught in the overlap between recovery and response, while facing compounding and cascading risks. In Sierra Leone, for example, the 2014–16 Ebola outbreak devastated health systems and livelihoods, leaving many survivors with lingering health complications and societal stigmatisation. These challenges were intensified by the impacts of a mudslide in Sierra Leone’s capital Freetown in 2017 that killed more than 1000 people and left thousands more homeless, particularly those living in vulnerable informal settlements.

Continuous states of anticipation and uncertainty also intersect with the non-linear temporalities of suffering, particularly among vulnerable groups, with gender playing a significant role in shaping impacts. Women and other marginalised populations can be disproportionately affected by the accretion of crises, and the care work that is ongoing across multiple timelines. For example, many women who were affected by Zika-related microcephaly in Brazil are the primary caregivers for children with multiple needs. These same communities were later particularly impacted by the effects of the COVID-19 pandemic. Now, in the same areas, there is preliminary evidence of possible vertical transmission in emerging Oropouche cases. WHO’s declaration of an end to the Zika-related microcephaly PHEIC did not mean an end for people living with Zika’s long-term impacts; in fact, it became harder for some communities to make their needs visible when other health issues were on the political agenda.

These protracted temporalities that play out in relation to situations of polycrisis are disproportionately affecting people in the Global South via socioeconomic, gendered, racialised, and geographical factors; and yet their temporal experiences of crises are generally inadequately funded and researched. Aiming to produce or declare an end without working to understand how endings are both constructed and experienced, and how people endure beyond them, is likely to produce not just unintended consequences, but predictable repetitions of injustice.

Examining the uncertain endings of epidemics in the past and present, from polio to mpox, underscores the need to rethink global health interventions in temporal terms. In an era marked by overlapping health emergencies in contexts of polycrisis, it is imperative to adopt a more inclusive and equitable framework that addresses the temporal experiences and needs of all populations. Building more equitable presents and futures will require focusing on the most marginalised people, shaping endings that reckon with the past through processes of reparative justice. For the past has not ended; it endures, sounding in the voice of lived experience that is asking to be heard.
